# Aspirin-Triggered Resolvin D1 Reduces Proliferation and the Neutrophil to Lymphocyte Ratio in a Mutant KRAS-Driven Lung Adenocarcinoma Model

**DOI:** 10.3390/cancers13133224

**Published:** 2021-06-28

**Authors:** Amanda Vannitamby, Mohamed I. Saad, Christian Aloe, Hao Wang, Beena Kumar, Ross Vlahos, Stavros Selemidis, Louis Irving, Daniel Steinfort, Brendan J. Jenkins, Steven Bozinovski

**Affiliations:** 1 School of Health & Biomedical Sciences, RMIT University, Bundoora 3083, Australia; amandavannitamby@gmail.com (A.V.); christian.aloe@rmit.edu.au (C.A.); hao.wang@rmit.edu.au (H.W.); ross.vlahos@rmit.edu.au (R.V.); stavros.selemidis@rmit.edu.au (S.S.); 2 Centre for Innate Immunity and Infectious Diseases, Hudson Institute of Medical Research, Clayton 3168, Australia; Mohamed.Saad@hudson.org.au (M.I.S.); brendan.jenkins@hudson.org.au (B.J.J.); 3 Department of Molecular Translational Science, School of Clinical Sciences, Monash University, Clayton 3168, Australia; 4 Department of Anatomical Pathology, Monash Health, Clayton 3168, Australia; Beena.Kumar@monashhealth.org; 5 Department of Respiratory Medicine, Royal Melbourne Hospital, Parkville 3050, Australia; Louis.irving@mh.org.au (L.I.); daniel.steinfort@mh.org.au (D.S.)

**Keywords:** lung cancer, adenocarcinoma, neutrophils, resolution, inflammation, ALOX5, FPR2, SAA, resolvin-D1, aspirin

## Abstract

**Simple Summary:**

Aspirin-triggered resolvin D1 (AT-RvD1) is biosynthesised by leukocytes as a mechanism to resolve inflammation during infection and/or injury. Emerging studies reveal that AT-RvD1 also has anti-cancer properties associated with stimulating macrophage-mediated clearance of tumour debris. No study to date has investigated how AT-RvD1 influences the neutrophil to lymphocyte ratio (NLR) in lung cancer, an established marker of poor prognosis. The biosynthesis of AT-RvD1 is dependent on the *ALOX5* gene, and we reveal that ALOX5 mRNA expression was markedly reduced in lung adenocarcinoma tumours. We next utilised an oncogenic *Kras^G12D^* lung adenocarcinoma mouse model to investigate the efficacy of AT-RvD1 in vivo. We show for the first time that AT-RvD1 reduces tumour growth in the lungs of *Kras^G12D^* mice and alters the immune landscape in tumours by reducing the NLR.

**Abstract:**

Tumour-associated neutrophils (TANs) can support tumour growth by suppressing cytotoxic lymphocytes. AT-RvD1 is an eicosanoid that can antagonise neutrophil trafficking instigated by ALX/FPR2 ligands such as serum amyloid A (SAA). We aimed to establish whether SAA and ALOX5 expression associates with TANs and investigate the immunomodulatory actions of AT-RvD1 in vivo. MPO-positive neutrophils were quantified in tumour blocks from lung adenocarcinoma (*n* = 48) and control tissue (*n* = 20) by IHC. Tumour expression of SAA and ALOX5 were analysed by RTqPCR and an oncogenic *Kras^G12D^* lung adenocarcinoma mouse model was used to investigate the in vivo efficacy of AT-RvD1 treatment. ALOX5 expression was markedly reduced in lung adenocarcinoma tumours. The SAA/ALOX5 ratio strongly correlated with TANs and was significantly increased in tumours harbouring an oncogenic *KRAS* mutation. AT-RvD1 treatment reduced tumour growth in *Kras^G12D^* mice, which was accompanied by suppressed cellular proliferation within parenchymal lesions. In addition, AT-RvD1 significantly reduced the neutrophil to lymphocyte ratio (NLR), an established prognostic marker of poor survival in adenocarcinoma. This study identifies a novel molecular signature whereby elevated levels of SAA relative to ALOX5 favour accumulation of TANs. Furthermore, the ALOX5/5-LO enzymatic product, AT-RvD1, markedly reduced the NLR and suppressed tumour growth in *Kras^G12D^* mice.

## 1. Introduction

Long-term low dose aspirin may reduce the risk of developing lung adenocarcinoma [1], although this association is poorly understood at a mechanistic level. Aspirin’s anti-inflammatory actions are elicited by blocking cyclooxygenase-2 (COX-2) synthesis of prostaglandins, which are implicated in angiogenesis, tumour metastasis and suppression of antitumour immunity [2]. Unfortunately, newer classes of COX-2 inhibitors such as celecoxib have failed to provide any survival advantage in NSCLC [3]. An intriguing explanation for this finding may be that aspirin displays a unique property whereby it can promote the biosynthesis of aspirin-triggered (AT) specialised pro-resolving mediators (SPMs) such as AT-resolvin D1 (AT-RvD1) [4].

AT-RvD1 is biosynthesised by a multistep process involving aspirin-acetylated COX2 and the 5-lipoxygenase (5-LO) enzyme encoded by the *ALOX5* gene. Both enzymes are expressed in leukocytes where aspirin-acetylated COX2 lipid metabolites are sequentially oxygenated by 5-LO, resulting in the production of AT-RvD1 [5]. Once synthesised, AT-RvD1 interacts with the ALX/formyl peptide receptor 2 (FPR2) on leukocytes to exert its pro-resolving functions that include antagonising neutrophil trafficking and stimulating macrophage efferocytosis [6,7]. It is also emerging that AT-resolvins display potent anti-cancer activity by promoting the clearance of therapy-generated tumour cell debris by macrophages [8]. Furthermore, the anti-tumour activity of aspirin is ALX/FPR2-dependent, as low dose aspirin failed to reduce tumour growth in ALX/FPR2 KO mice [9]. In contrast to AT-SPMs, alternative inflammatory ALX/FPR2 agonists such as serum amyloid A (SAA) may have a pathogenic role in lung cancer. SAA promotes inflammation by stimulating the recruitment of neutrophils into the lungs via an ALX/FPR2 dependant mechanism involving interleukin-17A [10,11].

SAA can also impair cytotoxic T cells functionality by promoting the expansion of IL-10-expressing neutrophils [12] and activating myeloid-derived suppressor cells [13]. Hence, ALX/FPR2 agonists have the potential to influence the neutrophil to lymphocyte ratio (NLR), which is a prognostic marker in NSCLC, where elevated NLR levels associate with poorer overall survival in NSCLC [14,15]. Tumour-associated neutrophils (TANs) are mainly detected within the tumour islet in adenocarcinoma [16] where they can switch their phenotype in response to the evolving molecular landscape [17]. TANs become more pathogenic as the tumour progresses [18], secreting proteases such as MMP9 [19] to support angiogenesis, proliferation, ECM remodelling and metastasis. In this current study, we identified a unique SAA^high^ALOX^low^ molecular signal or endotype that is associated with increased neutrophil infiltration within the tumour microenvironment in lung adenocarcinoma patients. In addition, we investigated the therapeutic efficacy of AT-RvD1 in vivo, which significantly reduced lung tumour growth in a mouse model genetically engineered to conditionally express the oncogenic *Kras^G12D^* allele. Our findings identify a novel avenue for therapeutically blocking pathogenic neutrophils in lung cancer.

## 2. Materials and Methods

### 2.1. Patient Characteristics

Surgically resected tumour tissue biopsies from patients with stage IA-IIIA NSCLC were obtained at the Royal Melbourne Hospital pathology department. A 1–2 cm^2^ sample of tumour tissue excess to diagnostic requirements was bisected with one piece snap frozen and one piece formalin fixed and paraffin embedded as previously described [20]. Specimens were archived by the Victorian Cancer Biobank with ethics approval (RMIT University Human Research Ethics Committee; Ethics ID: SEHAPP 09-17). Current guidelines for NSCLC classification [21] were used to classify tumour adenocarcinoma specimens (*n* = 48). Control lung tissue biopsies (*n* = 20) were obtained from patients without malignant disease (*n* = 10) and from the adjacent tumour-free resection tissue (*n* = 10) from a subset of the adenocarcinoma patients.

### 2.2. Immunohistochemical and Immunofluorescence Staining for Neutrophils, Macrophages and Lymphocytes

The following antibodies were used for the human tissue staining: rabbit polyclonal antibody against human MPO (Dako # A0398) and mouse monoclonal antibody against human Amyloid A (clone mc1; Dako # M0759). Serial control and tumour sections were submerged in preheated citrate buffer (10 mM Citric Acid, 0.05% Tween 20, pH 6.0) and incubated at 95 °C for 20 min using a Decloaking Chamber (Biocare Medical, Pacheco, CA, USA). Endogenous peroxidase activity was quenched using the dual enzyme block for 5 min (EnVision^®^ + Dual Link System-HRP kit, Agilent Technologies, Santa Clara, CA, USA). Slides were incubated for another 1 h at room temperature in blocking buffer specific for MPO (5% BSA, 10% Horse Serum, 0.4% TritonX in PBS) or SAA (5% HS, 0.4% TritonX in PBS). Sections were then incubated with MPO (1:500 dilution) or SAA (1:40 dilution) antibody in PBS for 1 h at room temperature. Following 3 washes, slides were incubated at room temperature with labelled polymer-HRP, supplied in the EnVision+ kit and washed three times prior to incubation with SIGMAFAST 3,3′-Diaminobenzidine (Sigma Aldrich, St. Louis, MO, USA) for 3–5 min. Sections were then counterstained in Mayer’s Haematoxylin (Trajan Scientific and Medical, Victoria, Australia), dehydrated and mounted onto slides using entellan mounting medium (Merck, Kenilworth, NJ, USA). To obtain an unbiased analysis of tumour-infiltrating immune cells with objective data, all immuno-stained slides were subjected to whole slide microscope scanning under high-power magnification using the Olympus V120 virtual slide scanner. The entire tumour section was captured and the area positive for MPO was determined using the Olympus CellSens software and presented as the area positive relative to the entire tumour section on the slide.

The following antibodies were used for the mouse tissue staining: goat polyclonal antibody against human MPO (R&D Systems # AF3667), rabbit monoclonal antibody against human CD3 (Abcam # ab16669), donkey polyclonal secondary antibody to goat IgG conjugated to Alexa Fluor 488 (Thermo Fisher Scientific # A32814) and donkey polyclonal secondary antibody to rabbit IgG conjugated to Alexa Fluor 568 (Thermo Fisher Scientific # A10042). Serial control and tumour sections of 5 μm thickness were deparaffinised in histolene and rehydrated in a series of graded ethanol steps. For heat-induced epitope retrieval, slides were submerged in preheated Tris-EDTA buffer (10 mM Tris base, 1 mM EDTA, 0.05% Tween 20, pH 9) and incubated at 95 °C for 20 min using a water bath. Slides were then rinsed in PBS and tissue permeabilised (0.5% Triton X-100 in PBS) for 10 min. Slides were next incubated in a humidity chamber for 1 h at room temperature in blocking buffer (5% BSA, 5% horse serum, 300 mM glycine in PBS-Tween 0.05%). Sections were then incubated overnight at 4 °C with MPO (1:40) and CD3 (1:40) antibodies in blocking buffer (no glycine). Following 3 washes (PBS with 0.05% Tween 20), slides were incubated at room temperature in secondary antibodies (1:200) in 1:10 diluted blocking buffer (no glycine). After 3 washes, slides were coverslipped with DAPI-fluorescence mounting medium (Thermo Fisher Scientific # 00-4959-52) and imaged under high power magnification using an Olympus BX53 upright microscope. Ten random high-power fields of the left lung were manually quantified for MPO- and CD3-positive cells.

### 2.3. TCGA and Oncolnc Database Analysis

TCGA datasets (PanCancer Atlas project) were used to analyse RNA-Sequencing data on 507 lung adenocarcinoma tumour specimens. cBioPortal (http://www.cbioportal.org, accessed date 2 June 2021, Memorial Sloan Kettering Cancer Center, New York, NY, USA) was used to extract the data and present ALOX5 and SAA1/2 mRNA expression as z-scores relative to normal/control samples. In addition, gene expression of ALOX5 and SAA were stratified according to mutation status of *KRAS*, *LKB1* and *TP53*. The OncoLnc database (www.oncolnc.org accessed date 2 June 2021) was utilised for Cox coefficient analysis between ALOX5 gene expression levels and survival in lung adenocarcinoma according to Kaplan-Meier estimates. The results are in whole or in part based on data generated by the TCGA Research Network (https://www.cancer.gov/tcga (accessed on 2 June 2021)).

### 2.4. Gene Expression Analysis by RTqPCR and Kras Genotyping

Total RNA and DNA was isolated from snap frozen biopsies using the AllPrep DNA/RNA kit (Qiagen, Hilden, Germany) according to the manufacturer’s instructions. The concentration of nucleic acid was determined using a NanoDrop spectrophotometer. Total RNA was converted to cDNA as previously described [19,20]. Quantitative PCR was performed on the QuantStudio 7 (Applied Biosystems, Carlsbad, CA, USA) using validated Taqman primer/probes to assess the transcript levels. Threshold cycle values (Ct) were normalised to the geomean of two stable reference genes TBP and PUM1 [20]. The comparative (2-ΔΔCt) method was used to present gene expression as fold change relative to a control sample as previously described [19,20]. SAA and ALOX5 expression were further analysed to generate four distinct groups (ALOX^LOW^SAA^LOW^, ALOX5^HIGH^SAA^LOW^, ALOX5^LOW^SAA^HIGH^, ALOX5^HIGH^/SAA^HIGH^). The 50th percentile was used as a cut-off to differentiate high versus low expression of ALOX5 and SAA. Kras mutation status was assessed using the frozen biopsy genomic DNA and the ddPCR Kras screening kit (BioRad, Hercules, CA, USA). The kit was used in conjunction with the QX200 Droplet Digital PCR System (BioRad, Hercules, CA, USA) according to the manufacturer’s instructions to screen for seven Kras mutations including G12A, G12C, G12D, G12R, G12S, G12V and G13D. A cut-off of 0.2% mutant allele frequency was used in accordance with the reported mutation detection cut-off value for this assay [22].

### 2.5. Quantification of SAA in Serum

The human SAA sandwich enzyme-linked immunosorbent assay (ELISA) kit (Thermo Fisher Scientific, Waltham, MA, USA) was used to quantify SAA levels in serum in accordance with the manufacturer’s instructions. The absorbance was measured at 450 nm using the CLARIOstar (BMG LABTECH, Ortenberg, Germany). The concentrations of the samples were determined using a 4-parameter logistic regression analysis of the standard curve.

### 2.6. Kras Mouse Model

The Kras^G12D^ strain (maintained on a C57BL/6 × 129Sv background) carrying a Lox-Stop-Lox (LSL) sequence followed by the Kras^G12D^ point mutation was used for this study. Lung adenocarcinoma was induced in 6-week-old male and female Kras^G12D^ mice by intranasal inhalation of 5 × 10^6^ plaque-forming units of Adenovirus Cre recombinase (Ad-Cre; University of Iowa) as previously described [23,24]. AT-RvD1 (Cayman chemicals, # 13060) or vehicle (10% ethanol in sterile saline) were initiated one day after Ad-Cre inhalation via intranasal inhalation (400 ng AT-RvD1 in a total volume 35 µL) twice weekly (Monday and Thursday) over 6 weeks. Mice were housed under specific pathogen-free conditions and were culled 6 weeks following Ad-Cre inhalation. Tumour burden, PCNA and TTF-1 levels in lungs of wildtype and *Kras^G12D^* mice were analysed as previously published [23,24]. All animal experiments were approved by the Monash University Medical Centre Animal Ethics Committees.

### 2.7. Statistical Analysis

Data analysis and graphs were generated using GraphPad Prism 7.02 (GraphPad Software Inc, San Diego, CA, USA). Mann–Whitney two-tailed *t*-tests were performed to compare two unpaired samples and the Wilcoxon matched-pairs signed rank test was used for paired analysis. A comparison of multiple groups was determined using the Kruskal–Wallis test followed by Dunn’s multiple comparison post-hoc tests. Data were presented as Tukey box plots. Spearman correlation was performed between two groups. Statistical significance for all analyses was determined by *p*-values less than 0.05.

## 3. Results

### 3.1. TANs Accumulate in Adenocarcinoma Biopsies and Correlate with SAA Expression

**Figure 1:**
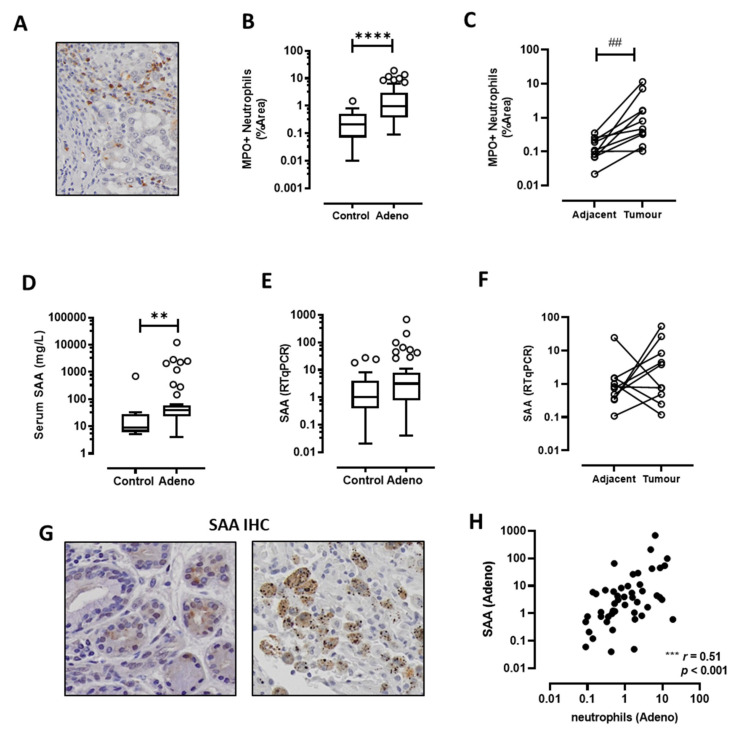
SAA levels positively correlate with tumour-associated neutrophil infiltration. (**A**) Representative images of MPO-positive neutrophils in a lung adenocarcinoma tumour section (original magnification ×200). (**B**) Neutrophils are significantly elevated in tumours compared to control tissue biopsies, which was confirmed by (**C**) paired analysis of tumour and adjacent control lung tissue. (**D**) Circulating SAA levels were elevated in lung adenocarcinoma patients as determined by ELISA. (**E**) SAA transcript levels were not increased in tumours as measured by RTqPCR and confirmed by (**F**) sub-analysis of the paired from the tumour and adjacent control tissue samples. (**G**) Tissue sections were stained for SAA, which identified positive staining within seromucinous glands and tumour-associated macrophages (original magnification ×200). (**H**) Spearman correlation was used to assess associations between SAA transcript levels and tumour infiltrating neutrophil density (*r* = 0.51, *** *p* < 0.001). **** *p* < 0.0001, ** *p* < 0.01, ## *p* < 0.01.

MPO-positive neutrophils were detected throughout the tumour section (Figure 1A) and there was a significant increase in neutrophil infiltration compared to the control biopsies (Figure 1B). Paired sub-analysis of matching biopsies from the tumour and adjacent control tissue confirmed that neutrophils accumulated in greater number within the tumour itself (Figure 1C). Since SAA is implicated in the recruitment of neutrophils into the lungs, SAA levels in serum and tissue were analysed. Circulating levels of serum SAA were significantly increased in lung adenocarcinoma patients compared to non-malignant control patients (Figure 1D). However, SAA mRNA expression was not significantly altered in tumour compared to control tissue in either the unpaired (Figure 1E) or paired analysis (Figure 1F). IHC was performed to identify the local sources of SAA and images were evaluated by an experienced pathologist, who identified SAA positive staining within seromucinous glands and cells consistent with the morphology of macrophages (Figure 1G). Whilst SAA mRNA levels were not significantly increased in tumours, there was a strong positive association with neutrophils (Figure 1H, *r* = 0.51, *p* < 001).

### 3.2. The SAA/ALOX5 Ratio Is Increased in KRAS Mutated Lung Adenocarcinoma

**Figure 2:**
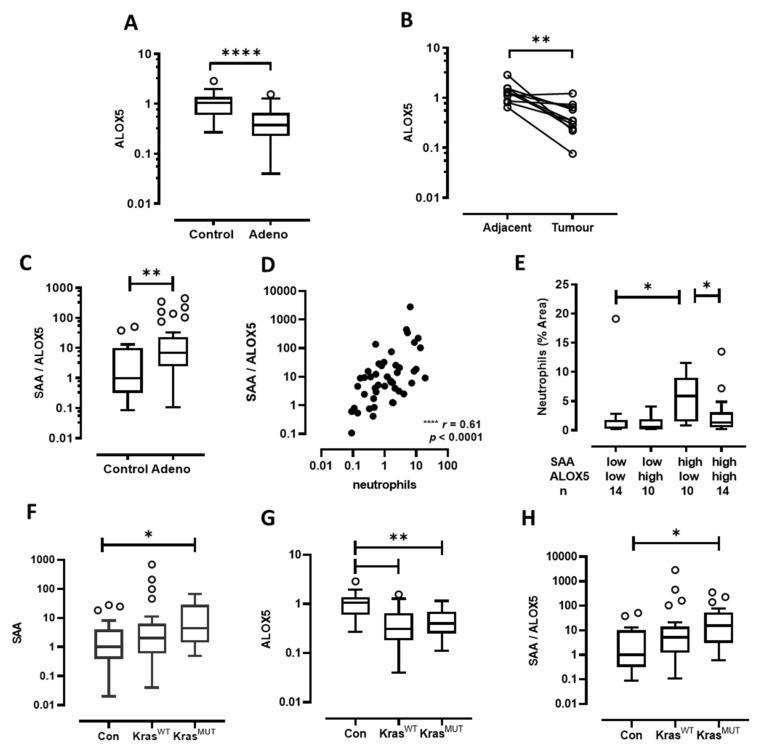
The SAA/ALOX5 ratio is elevated in lung adenocarcinoma. (**A**) ALOX5 transcript levels were significantly reduced in lung adenocarcinoma tumour biopsies as assessed by RTqPCR, which was confirmed by (**B**) paired analysis tumour and adjacent control samples. (**C**) The SAA/ALOX5 ratio was significantly increased in tumour biopsies and (**D**) Spearman correlation identified a strong positive association between the SAA/ALOX5 ratio and tumour infiltrating neutrophils (*r* = 0.61, *p* < 0.0001). (**E**) The adenocarcinoma cohort was stratified into four groups based on their upper and lower SAA and ALOX5 expression levels (50th percentile cut-off). (**F**) SAA expression was assessed according to *KRAS* mutation status. (**G**) ALOX5 expression was assessed according to *KRAS* mutation status. (**H**) The SAA/ALOX5 ratio according to *KRAS* mutation status. **** *p* < 0.0001, ** *p* < 0.01, * *p* < 0.05.

In contrast to SAA, ALOX5 transcript expression was significantly reduced in adenocarcinoma tissue biopsies in both the unpaired and paired analysis (Figure 2A,B). The expression levels of SAA relative to ALOX5 (SAA/ALOX5 ratio) were next assessed using the same biospecimens. The SAA/ALOX5 ratio was significantly increased in adenocarcinoma compared to control biopsies (Figure 2C) and this ratio strongly correlated with MPO-positive neutrophils in adenocarcinoma (Figure 2D). This cohort was next stratified based on SAA and ALOX5 mRNA expression to generate four distinct groups (ALOX^LOW^SAA^LOW^, ALOX5^HIGH^SAA^LOW^, ALOX5^LOW^SAA^HIGH^, ALOX5^HIGH^/AA^HIGH^) where there was a relatively even distribution of patient numbers (Figure 2E). MPO-positive neutrophils were compared across these four groups, identifying significantly elevated neutrophil infiltration in SAA^HIGH^ALOX5^LOW^ tumours (Figure 2E). Importantly, neutrophil infiltration was significantly reduced in SAA^HIGH^ALOX5^HIGH^ tumours (Figure 2E), to suggest that increased expression of ALOX5 in tumours may enhance SPM production to oppose the inflammatory actions of SAA.

Tumour biopsies were then genotyped for oncogenic *KRAS* mutations by ddPCR. Using a cut-off of 0.2% mutant allele frequency, which is the reported reliable mutation detection cut-off for this assay [22], 17/48 (35%) of the tumours were classified as harbouring an activating *KRAS* mutation. The majority of *KRAS* mutated patients (80%) had a history of cigarette smoking, which is consistent with studies that have identified smoking as a risk factor for acquiring activating *KRAS* mutations [25]. The expression of SAA transcript was evaluated according to *KRAS* status, where levels were significantly increased in *KRAS* mutated but not wild-type tumour biopsies (Figure 2F). In addition, the expression of ALOX5 transcript was assessed, where ALOX5 levels were decreased irrespective of *KRAS* status (Figure 2G). Increased SAA expression and decreased ALOX5 expression resulted in a significant increase in the SAA/ALOX5 ratio in *KRAS* mutated tumours but not in *KRAS* wild-type tumours (Figure 2H). The frequency of SAA^HIGH^ALOX5^LOW^ patients was twofold higher in *KRAS* mutated tumours compared to *KRAS* wild-type tumours (5/17 = 29% vs. 5/31 = 16% respectively). Taken together, these observations suggest that acquisition of the *KRAS* driver mutation represents a molecular abnormality that will drive increased SAA expression in a tumour microenvironment where ALOX5 expression is reduced. SAA^HIGH^ALOX5^LOW^ tumours harbouring a wild-type *KRAS* genotype were also observed, albeit at a lower frequency, to suggest that this endotype is not restricted to *KRAS* mutated patients.

**Figure 3:**
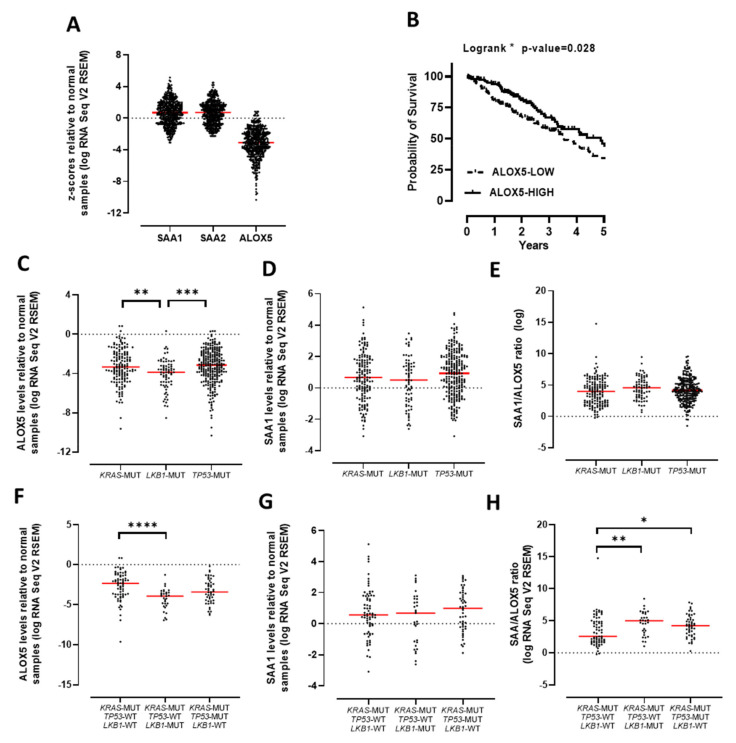
TCGA dataset analysis validate that ALOX5 expression is reduced in lung tumours. (**A**) SAA1, SAA2 and ALOX5 gene expression derived from the TCGA RNA Seq lung adenocarcinoma dataset were expressed relative to normal tissue. (**B**) Kaplan–Meier plot of overall survival for lung adenocarcinoma patients stratified by low (lower 33rd percentile) and high (upper 33rd percentile) ALOX5 expression (data from OncoLnc). (**C**) ALOX5 gene expression stratified into KRAS, TP53 or LKB1 mutated (MUT) tumours. (**D**) SAA1 gene expression stratified into KRAS, TP53 or LKB1 mutated tumours. (**E**) The SAA1/ALOX5 ratio presented according to tumour genotype. (**F**) ALOX5 levels relative to normal samples. (**G**) SAA1 levels relative to normal samples. (**H**) SAA/ALOX5 ratio. **** *p* < 0.0001, *** *p* < 0.001, ** *p* < 0.01, * *p* < 0.05.

In order to validate SAA and ALOX5 gene expression, publicly available TCGA RNA Seq datasets were analysed. Gene expression z-scores for SAA1/2 and ALOX5 were presented relative to normal tissue. Consistent with our dataset, there was a broad range of SAA1/2 expression levels in lung adenocarcinoma tumours, including many tumours with high SAA levels (Figure 3A). However, median levels were not significantly different from normal tissue. Analysis of ALOX5 gene expression in this TCGA dataset was also very consistent with our findings, where ALOX5 levels were uniformly reduced in tumours relative to normal tissue (Figure 3A). The OncoLnc database provides a platform for the assessment of correlations between survival and gene expression derived from TCGA. This analysis demonstrates that ALOX^LOW^ patients displayed a significantly reduced survival advantage when compared to ALOX^HIGH^ patients (Figure 3B). The TCGA dataset was further stratified based on the mutational status of the tumours focusing on the three most common mutations found in lung adenocarcinomas. This analysis demonstrates that ALOX5 expression is reduced in *KRAS*, *TP53* and *LKB1* mutated tumours, where levels were particularly reduced in *LKB1* mutated tumours (Figure 3C). SAA1 expression was not significantly different in tumours harbouring a *KRAS*, *TP53* or *LKB1* mutation, where a large proportional of tumours expressed high levels of SAA mRNA relative to normal tissue irrespective of genotype (Figure 3D). We next analysed the SAA/ALOX5 ratio and observed an increased ratio of SAA relative to ALOX5 mRNA levels irrespective of tumour genotype (Figure 3E). Expression was next evaluated in tumours harbouring multiple mutations, where ALOX5 expression was significantly reduced in tumours with a *KRAS* and *LKB1* mutation (Figure 3F). SAA expression was not altered; however, the *SAA*/*ALOX5* ratio was significantly higher in *KRAS*/*LKB1* and *KRAS*/*TP53* double-mutant tumours (Figure 3G,H).

### 3.3. AT-RvD1 Reduces Tumour Growth and the NLR in Kras^G12D^-Induced Lung Adenocarcinoma

**Figure 4:**
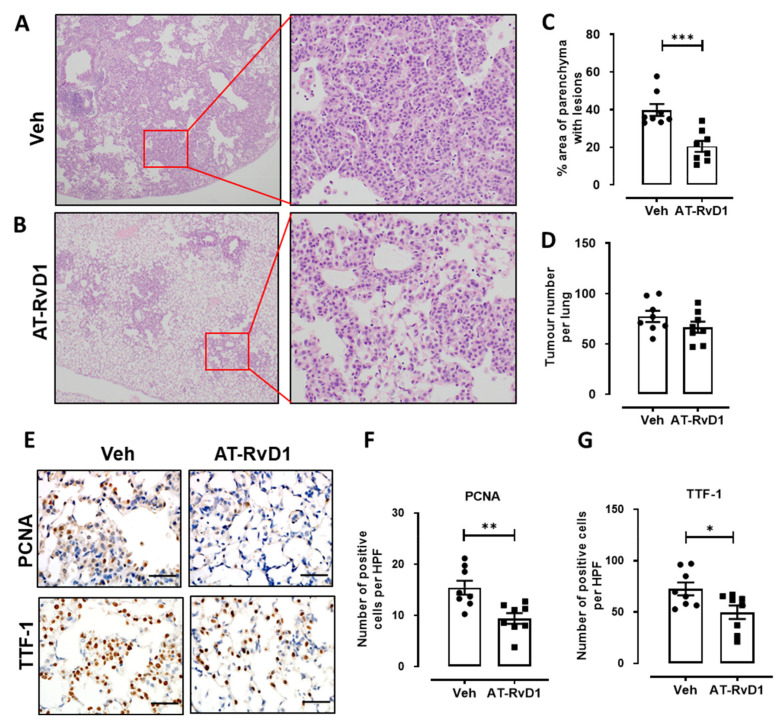
AT-RvD1 reduced tumour burden in *Kras^G12D^* mice. (**A**) Representative images of parenchymal lesions that develop in response to adeno-CRE delivery following 6 weeks of induction in vehicle treated mice (original magnification, ×40 & ×100). (**B**) Representative images of parenchymal lesions that develop in response to adeno-CRE delivery (original magnification, ×40 & ×200) following 6 weeks of induction in AT-RvD1 treated mice (400ng, 2× weekly). (**C**) Quantification of lung parenchyma area occupied by tumour lesions and (**D**) quantification of tumour incidence per whole lung across the treatment groups. (**E**) Representative images of PCNA and TTF-1 stained lung sections (original magnification, ×200) and (**F**) quantification of PCNA positive cells per high powered field in lungs of the indicated treatment groups. (**G**) Quantification of TTF-1 positive cells per high powered field in lungs of the indicated treatment groups. *n* = 8 per group, *** *p* < 0.0005, ** *p* < 0.01, * *p* < 0.05 unpaired *t*-test.

Since ALOX5 is reduced in lung adenocarcinoma and required for AT-RvD1 production, we next evaluated the therapeutic potential of this SPM in the well-established *Kras^G12D^* lung adenocarcinoma model. At 6 weeks post-induction of the oncogenic *Kras^G12D^* allele by Ad-Cre inhalation, *Kras^G12D^* mice displayed diffuse atypical adenomatous hyperplasia (AAH), adenoma and sporadic adenocarcinoma in situ (AIS) lesions throughout the lung parenchyma (Figure 4A), which is consistent with previous studies from our group and other groups using this model [23,26,27]. Approximately 40% of the lung parenchyma contained lesions in vehicle-treated *Kras^G12D^* mice, which is similar to our previous findings [23], and the area of parenchymal lesions was significantly reduced by approximately 50% in AT-RvD1-treated mice (Figure 4A–C). The number of lesions were not significantly different between vehicle and AT-RvD1 treated mice (Figure 4D), suggesting that the growth of lesions was suppressed. To explore this further, the proliferative index of tumour-bearing *Kras^G12D^* mice was assessed by quantifying PCNA levels by IHC, which were significantly reduced in AT-RvD1 treated mice by approximately 40% (Figure 4E,F). In addition, the number of cells positive for the lung adenocarcinoma marker thyroid transcription factor-1 (TTF-1) were significantly reduced by 32% with AT-RvD1 treatment (Figure 4E,G).

**Figure 5:**
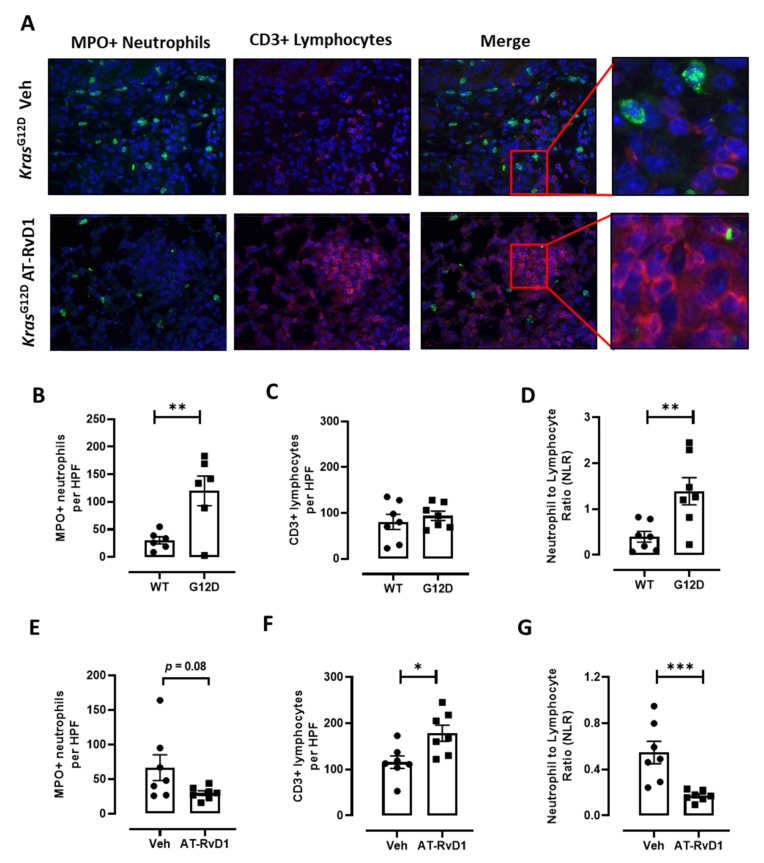
AT-RvD1 reduces the neutrophil to lymphocyte ratio. (**A**) Representative immunofluorescent images of MPO-positive neutrophils, CD3-positive lymphocytes and merged images (right panels) from *Kras^G12D^* mice that were intranasally treated with AT-RvD1 (400 ng, 2× weekly) or vehicle over a 6-week period following Ad-Cre inhalation (original magnification ×200). (**B**) Quantification of MPO-positive and (**C**) CD3-positive cells per high powered field in lungs of the indicated genotype. (**D**) The neutrophil to lymphocyte (NLR) was determined for the same group of mice (NLR). *n* = 7 per group, ** *p* < 0.001, * *p* < 0.05 unpaired *t* test. Quantification of (**E**) MPO-positive and (**F**) CD3-positive cells HPF in lungs of *Kras^G12D^* mice treated with AT-RVD1 or vehicle. (**G**) The neutrophil to lymphocyte ratio (NLR). *n* = 7 per group, *** *p* < 0.0005, * *p* < 0.05 unpaired *t* test.

We next quantified MPO-positive neutrophils and CD3-positive lymphocytes by immunofluorescence imaging. Neutrophils and lymphocytes were detected throughout the lungs of *Kras^G12D^* mice (Figure 5A) and there was a significant increase in neutrophil infiltration in the lungs of mice harbouring the *Kras^G12D^* genotype compared to wild-type mice (Figure 5B). In contrast, the number of CD3-positive lymphocytes was not altered in *Kras^G12D^* mice compared to wild-type mice (Figure 5C). The NLR in tumour biopsies was also determined, which showed that the NLR was significantly elevated in *Kras^G12D^* mice compared to wild-type mice (Figure 5D). We next evaluated whether AT-RvD1 altered neutrophil and lymphocyte numbers in the lungs of *Kras^G12D^* mice. The mean number of MPO-positive neutrophils was reduced with AT-RvD1 treatment; however, this reduction was not significant (Figure 5E, *p* = 0.08 Veh vs. AT-RvD1). There was a significant increase in the number of CD3-positive lymphocytes in the lungs of *Kras^G12D^* mice treated with AT-RvD1 (Figure 5F). Furthermore, there was a marked reduction in the NLR in the lungs of AT-RvD1 treated mice compared to wild-type mice (Figure 5G).

## 4. Discussion

In this study, ALOX5 expression was markedly reduced in lung adenocarcinoma tumours and this finding was reproducibly observed in the TCGA RNA Seq datasets. This is an intriguing finding as 5-LO enzymatic activity is traditionally viewed as a pro-inflammatory and potentially pro-tumourigenic mediator through the synthesis of leukotrienes. There are multiple studies that implicate 5-LO activity in tumour cell proliferation and survival, and 5-LO expression levels have been reported to be increased in pancreatic, prostate and breast cancer (as reviewed in [28]). Because of this, there has been interest in developing 5-LO inhibitors; however, cytotoxic off-target effects raise concern about their clinical application [28]. Our findings raise further doubts about pharmacological strategies that inhibit 5-LO activity for the treatment of lung cancer, as ALOX5 expression is already significantly reduced in lung tumours.

Leukocytes normally express high levels of ALOX5/5-LO and lung tumours are enriched for leukocytes. Hence, the tumour microenvironment is likely reducing ALOX5 mRNA in leukocytes. We investigated whether common mutations (*KRAS*, *TP53* or *LKB1*) can influence ALOX5 expression in tumour biospecimens. Using the large TCGA datasets, ALOX5 was reduced irrespective of tumour genotype with lowest expression observed in *KRAS*/*LKB1* mutated tumours. LKB1 is encoded by the *STK11* gene that is frequently altered in lung adenocarcinomas. *STK11* mutations cause LKB1 protein truncation and loss of tumour suppressive function. *LKB1* levels can also be reduced by promoter hypermethylation [29]. The *ALOX5* gene is also altered by methylation as its promoter region contains eight proximal GC-boxes [30]. Promoter hypermethylation significantly reduces ALOX5 expression in leukocyte cell-lines [31]. It is also established that DNA methylation signatures are markedly altered in blood leukocytes of heavy smokers and lung cancer patients [32]. It is plausible that promoter methylation is responsible for reducing both *LKB1* and *ALOX5* levels in the lung cancer tumour microenvironment, although further work is needed to confirm this.

We propose that a secondary consequence of reduced ALOX5 expression in lung adenocarcinoma is that the production of SPMs such as RvD1 and AT-RvD1 will also be reduced in the tumour microenvironment. This concept is supported by the finding that ALOX5/5-LO appears to have a tumour suppressive role in lung cancer, as global genetic deletion of *ALOX5* resulted in substantially increased lung tumour growth in a mouse model where Lewis lung carcinoma cells were implanted directly into the lungs [33]. The deletion of *ALOX5* in this model resulted in reduced leukotriene production; however, levels of alternative lipid metabolites such as SPMs were not measured [33]. We did not directly measure these lipid metabolites in our archived tumour biospecimens as they are unstable metabolites that require specialised tissue processing. However, we did measure an alternative biological readout, namely expression of ALOX5, which is essential for biosynthesis of SPMs such as RvD1. Both RvD1 and AT-RvD1 trigger the resolution of inflammation by interacting with the ALX/FPR2 receptor. ALX/FPR2 is a complex G-protein–coupled receptor (GPCR) capable of interacting with a diverse array of agonists that can initiate contrasting biological signals. The agonists fall into two broad categories including pro-resolving mediators such as AT-RvD1 and pro-inflammatory agonists such as SAA [34,35]. Alternative agonists classes are proposed to interact with different regions within the ALX/FPR2 receptor to initiate distinct conformational changes that trigger contrasting signalling. For example, when SAA binds to ALX/FPR2, it promotes leukocyte trafficking and delays neutrophil apoptosis, whereas AT-RvD1 suppresses leukocyte trafficking and promotes neutrophil apoptosis [34,35].

SAA can also support tumour growth by activating neutrophils and myeloid-derived suppressor cells [12,13]. An imbalance in the levels of SAA relative to SPMs such as AT-RvD1 that target the ALX/FPR2 receptor has been proposed to occur in lung cancer but not formally proven [6]. Our study demonstrates for the first time that there is an imbalance in SAA relative to ALOX5 and that a high SAA/ALOX5 ratio is strongly associated with neutrophilic inflammation in lung tumours. This finding is consistent with the known chemotactic activity of SAA, which stimulates neutrophil migration into the lungs via an ALX/FPR2 receptor dependant mechanism [10,11,36]. Furthermore, we investigated the cellular source of SAA in the lung tumour microenvironment by IHC staining. Macrophages were identified as a potential source of SAA, but co-staining with SAA and a macrophage specific marker such as CD68 is required to confirm this finding. Consistent with our observation, macrophages have been identified as a main source of SAA in the tumour microenvironment of breast cancer [37]. Higher SAA expression was detected in tumours that harboured oncogenic *KRAS* activating mutations compared to control tissue in our study. Further analyses of the larger TCGA dataset revealed that a high SAA/ALOX5 ratio in tumours is not exclusive to *KRAS* mutated tumours, as high levels were detected in *TP53* and *LKB1* mutated tumours. We did identify a strong positive association between SAA and IL6 expression in tumour biospecimens (Spearman *r* = 0.55, *p* < 0.0001). Our finding is highly consistent with the known biology of the IL-6/STAT3 pathway, which is essential for maximal expression of expression of SAA [38]. Since IHC analysis did not identify tumour cells as a source of SAA, the acquisition of mutations such as *KRAS* does not appear to directly stimulate increased expression of SAA by transformed tumour cells. Lung tumours can be intrinsically pro-inflammatory with increased IL-6 levels driving signalling pathways that support carcinogenesis [23,39]. We propose that transformed cancer cells produce inflammatory cytokines such as IL-6, which act in a paracrine fashion on resident lung macrophages to produce SAA.

There are multiple recent studies demonstrating the tumour suppressive actions of SPMs such as AT-RvD1, which support its progression into the clinic. These studies show that AT-RvD1 acts via the ALX/FPR2 receptor expressed on leukocytes to reduce tumour growth. For example, RvD1 acts via ALX/FPR2 on macrophages to reduce tumour growth by promoting clearance of tumour cell debris and inhibiting macrophage-secreted proinflammatory cytokines [8,9]. SPMs that interact with ALX/FPR2 can also regulate the phenotype of tumour-associated macrophages, whereby SAA polarised macrophages support tumour growth, which was suppressed by SPMs [40]. RvD1 can also reduce cancer growth in a manner that was dependent on neutrophils, as depleting neutrophils with an anti-Ly6G antibody markedly suppressed the anti-tumour actions of RvD1 in vivo [41]. They propose that RvD1 reprograms neutrophils to recruit classical monocytes that display anti-cancer actions. Our study is the first to utilise the *Kras^G12D^* mouse model of lung adenocarcinoma to evaluate whether AT-RvD1 has anti-tumour properties in this pre-clinical model. We demonstrate that AT-RvD1 effectively reduced growth and proliferation of transformed cells as assessed by quantifying the area of parenchymal lesions and PCNA staining. It is also the first study to determine whether AT-RvD1 can alter the neutrophil to lymphocyte ratio, a prognostic marker of poor survival in NSCLC [14,15]. We focused on this outcome as TANs can suppress the function of cytotoxic lymphocytes. For example, neutrophil-generated reactive oxygen species (ROS) can suppress the metabolic switch in activated lymphocytes that is required for clonal T cell expansion and cytokine production [42]. Moreover, the expression of arginase-1 by neutrophils correlates with poor prognosis in NSCLC, and can suppress T cell function by depleting L-arginine required for T cell proliferation [43]. We demonstrate that AT-RvD1 markedly suppresses the NLR ratio in oncogenic *Kras* lung tumours and propose that the suppression of pathogenic neutrophils by AT-RvD1 promotes a more robust anti-tumour lymphocytic response.

## 5. Conclusions

We have identified that a subset of lung adenocarcinoma patients express higher levels of SAA relative to ALOX5 and that the SAA/ALOX5 ratio positively correlates with neutrophil infiltration. Our findings support the clinical application of AT-SPMs such as AT-RvD1 in lung cancer, whereby AT-RvD1 potently suppressed tumour growth in *Kras^G12D^* mice. A limitation of our study is that AT-RvD1 was delivered immediately after the initiation of *Kras* transformation and that lesions were assessed at 6 weeks post-induction, where in situ adenocarcinomas are sporadic. Future studies should evaluate the efficacy of AT-RvD1 in the therapeutic setting where it is delivered to tumours that are already established and more advanced. This study does provide important preclinical proof-of-concept that AT-RvD1 can be further developed as a treatment approach for future clinical implementation. We also demonstrate for the first time that the NLR is elevated in parenchymal lesions of *Kras^G12D^* mice and AT-RvD1 significantly reduced the NLR. Hence, we propose that AT-RvD1 can suppress the accumulation of TANs that are capable of inhibiting cytotoxic lymphocyte function within tumours. Future studies should focus further on comprehensively phenotyping neutrophils in the tumour microenvironment and establishing how AT-SPMs influence the phenotype of tumour-associated myeloid cells.

## Data Availability

All data are contained within this article. The lung cancer TCGA dataset analysed in this study is publicly available in the GDC Data Portal (https://portal.gdc.cancer.gov/), accessed on 2 June 2021.
